# A mathematical representation of the reactive scope model

**DOI:** 10.1007/s00285-023-01983-9

**Published:** 2023-08-30

**Authors:** Justin Wright, Kelly Buch, Ursula K. Beattie, Brenna M. G. Gormally, L. Michael Romero, Nina Fefferman

**Affiliations:** 1 Department of Ecology and Evolutionary Biology, University of Tennessee Knoxville, 569 Dabney, Knoxville, 37996 TN USA; 2 National Institute of Mathematical and Biological Synthesis, Knoxville, TN 37996 USA; 3 Department of Mathematics and Statistics, Austin Peay State University, Maynard Mathematics and Computer Science Building Room 205, Clarksville, TN 37044 USA; 4 Department of Biology, Tufts University, 200 Boston Ave #4700, Medford, MA 02155 USA

**Keywords:** Reactive scope, Stress, Stress schedule, Homeostasis, Senescence, 92C30

## Abstract

Researchers have long sought to understand and predict an animal’s response to stressful stimuli. Since the introduction of the concept of homeostasis, a variety of model frameworks have been proposed to describe what is necessary for an animal to remain within this stable physiological state and the ramifications of leaving it. Romero et al. (Horm Behav 55(3):375–389, 2009) introduced the reactive scope model to provide a novel conceptual framework for the stress response that assumes an animal’s ability to tolerate a stressful stimulus may degrade over time in response to the stimulus. We provide a mathematical formulation for the reactive scope model using a system of ordinary differential equations and show that this model is capable of recreating existing experimental data. We also provide an experimental method that may be used to verify the model as well as several potential additions to the model. If future experimentation provides the necessary data to estimate the model’s parameters, the model presented here may be used to make quantitative predictions about physiological mediator levels during a stress response and predict the onset of homeostatic overload.

## Introduction

According to Levine (2005), references to the idea of *homeostasis* and its disruption, which is often referred to now as *stress*, can be traced at least as far back as early Greek physicians and our modern perspectives on stress can largely be traced back to Cannon (1932) and Selye (1946). Attempts to provide a well-defined and widely-accepted definition for stress in animals have been ongoing for nearly as long with much of the ambiguity coming from the cyclical nature of defining a stimulus as stressor if it elicits a stress response and a stress response as a physiological or behavioral response to a stressor (Levine 2005; Romero et al. 2009). More recently, Sterling and Eyer (1988) proposed the allostasis model in an effort to redefine the concept of stress in terms of energy acquisition and usage. In turn, McEwen and Wingfield (2003) used the concept of allostasis to explain physiological mediator levels, such as glucocorticosteroids, through ecological factors like foraging success, bad weather, and habitat changes.

Romero et al. (2009) introduced the reactive scope model to both further the allostasis model while also addressing its weaknesses. Unlike the allostasis model, which focuses heavily on resource allocation and expenditure, the reactive scope model allows the concept of stress or a stress event to be broadly interpreted allowing for the consideration of changes to homeostasis or even anticipation of changes to homeostasis. Broadly speaking, the reactive scope model introduces two novel components to the consideration of stress. First, the concept of a *stressor* or *stress event*, a stimuli which causes stress, is allowed to include responses in anticipation of an environmental stressor. Second is the addition of the eponymous *reactive homeostasis* range to the traditional homeostasis model. The reactive homeostasis range is believed to exist as a dynamic buffer zone representing temporary resilience between traditional homeostasis and homeostatic overload which shrinks, temporarily or permanently, when an animal is experiencing a stress event causing mediator levels outside of homeostasis. The authors use the term “wear and tear” to refer to the shrinking of the reactive homeostasis range due to the accumulated cost of maintaining the stress response. The authors also point out that the use of “wear and tear” should not be, in this case, associated with the slow accumulation of damage and aging.

Since the introduction of the reactive scope model, many studies have interpreted results in terms of the model (Aguilar et al. 2016; Beattie et al. 2023a; Charpentier et al. 2018; Crespi et al. 2013; Eguizábal et al. 2022; Gabriel et al. 2018; Houtz et al. 2022; Howell and Sanchez 2011; Leishman et al. 2022; Lima et al. 2022; Pahuja and Narayan 2021; Roast et al. 2019; Romero 2012; Schoenle et al. 2018 to name a few). On the other hand, a relative few studies, including Gormally et al. (2019b), Beattie et al. (2023b), Gormally et al. (2019a), DuRant et al. (2016b), and DuRant et al. (2016a) have set out with the specific goal of verifying the reactive scope model with varying degrees of success. Generally, these studies caused stress reactions in groups of wild-caught birds, allowed the groups to recover for varying lengths of time, then induced new stress reactions with the hypothesis that groups with longer recovery times would have enough time to recover from reactive homeostasis allowing for a reduced stress response in comparison to groups with shorter recovery times. However, since the current version of the reactive scope model is purely conceptual, there was no way for these researchers to determine the necessary duration of stress events or recovery to ensure the hypothesized wear and tear would be observable.

As Grindstaff et al. (2022), Servedio et al. (2014), and Zavala et al. (2019) have pointed out, mathematical models can be powerful tool in the investigation theoretical biological and endocrinological theory. In Luttbeg et al. (2021), Taborsky et al. (2021), and Taborsky et al. (2022), the authors have used mathematical models to explore the evolution of the stress response and how repair rates influence basal levels. Mathematical frameworks and models are already being utilized in other investigations of the hypo-pituitary-adrenal (HPA) axis, stress, and the stress response. Using mathematical models to investigate the HPA axis itself has garnered a great deal of interest since the seminal work provided by Dempsher et al. (1984). Reviews of the subject have been offered by Lightman (2008), Goel et al. (2014), Nicolaides et al. (2017), Tsigos and Chrousos (2002), Lightman and Conway-Campbell (2010), Vinther et al. (2011), Gudmand-Hoeyer et al. (2014), Spiga et al. (2015), Zavala et al. (2019) and Hosseinichimeh et al. (2015). Hosseinichimeh et al. (2015) in particular offers a comparison of 14 published models and attempts to fit them to data with varying results and then goes on to provide an updated form of the most promising model. Since these reviews, others, such as Rao and Androulakis (2019) and Stanojević et al. (2018), have continued efforts to explore these models and discuss progress. The models constructed thus far have sought to capture the complex nature of the HPA axis made up of a system of molecular interactions, reaction-transport processes, and pathways using either systems of ordinary differential equations or delay differential equations that describe both basal and stress induces cortisol levels in humans or corticosterone levels in rats. However, as Stanojević et al. (2018) points out, while the field of mathematical modeling of the HPA axis is rich and growing, “there is still neither consensus nor a common representation of the core feedback mechanisms.” In addition, the models have often been constructed and calibrated with particular data sets and the models’ predictions are not tested across other published data sets. Further, while the analysis of these models has included in silico experimentation with acute stress events that perturb the system being described, they have not explored the degradation of the system’s ability to cope with prolonged or severe stress. These models may also have little to no application outside of the species for which they were designed and the molecules involved in the stress response process may differ among species (Taborsky et al. 2021). Finally, while existing models of the HPA axis are simplifications of the complex system, they are potentially unapproachable to researchers whose backgrounds do not include a significant amount of mathematics (Fawcett and Higginson 2012).

One weakness of the reactive scope model in its current formulation is that it is a conceptual framework presented graphically. That is, to date there has been no quantitative representation of the reactive scope model provided. As a consequence, there has been no way to predict experimental outcomes leaving no way to objectively verify the model. As pointed in by Grindstaff et al. (2022), the formulation of a mathematical model cannot, on its own, verify the reactive scope model. Confirming the conceptual model will require the combined work of empiricists and mechanistic models like the one presented here. With a mathematical model describing the reactive scope model, the parameters of the mathematical model can be estimated using regression techniques and experimental data, then the model may be used to predict physiological mediator levels in particular circumstances and this prediction can in turn be compared to experimental data. If the model and data are found to be in good agreement, we may presume that the mathematical model accurately captures the stress response process. If not, then the model can be adjusted as needed.

We provide a quantitative framework for the reactive scope model as well as several of its components. We begin describing general stress events and representing those stress events mathematically as a function of time in Sect. 2.1. In Sect. 2.2 we provide a time-dependent form for mediator levels subject to circadian and seasonal variation and then describe the reactive scope model as a system of ordinary differential equations that describe the boundaries between the ranges in the reactive scope model. We proceed then to provide several examples of the flexibility of this mathematical model. In Sect. 3 we use the mathematical model to duplicate the results of an empirical study and provide a broad analysis of the mathematical model. We provide a method to determine the veracity of the model empirically in Sect. 4. Finally, in Sect. 5.1, we provide several possible additions to the reactive scope model and discuss its weaknesses.

## The model

**Fig. 1:**
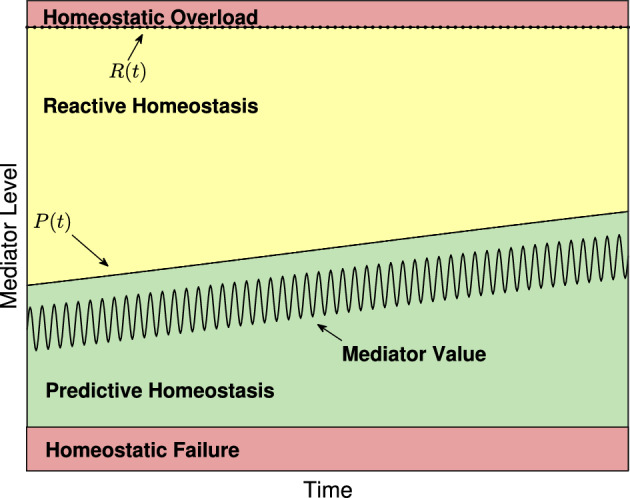
A generic example of mediator levels in the reactive scope model and of the system in (8) with no stress event used to duplicate figures depicting reactive scope given by Romero et al. (2009). Note that the size of the reactive homeostasis range (the distance between *R*(*t*) and *P*(*t*)) is shrinking due to a long-term, seasonal variation in the size of the predictive homeostasis range. The fluctuations in the mediator value are meant to mimic circadian variation

Fischer and Romero (2013), McEwen (2003), McEwen and Wingfield (2003), and Romero et al. (2009) all seek to describe the effects of stress on physiological mediators such as glucocorticoids, antibody titers, catecholamines, and heart rate to name a few. In the reactive scope model, Romero et al. (2009) hypothesized that a physiological mediator, such as changes in behavior, mediators of immune function, and mediators of the HPA axis as presented by McEwen (2003) and McEwen and Wingfield (2003), to be in one of four classifications based on range: predictive homeostasis, reactive homeostasis, homeostatic overload, and homeostatic failure. We adopt the same terminology here. Mediator values are in the *predictive homeostasis* range when an animal is able compensate for any normal fluctuations caused by seasonal and circadian changes as well as normal foraging behavior as might be associated with allostasis. Romero et al. (2009) introduced the *reactive homeostasis* range. Mediator values in the reactive homeostasis range are beyond the normal fluctuations represented by the predictive homeostasis range but are still low enough to temporarily avoid entering homeostatic overload. The terms predictive homeostasis and reactive homeostasis are borrowed from Moore-Ede (1986) and together they represent the normal reactive scope for an animal. Mediator levels that exceed the reactive homeostasis range are said to be in the *homeostatic overload* range and will induce a pathological state. The final range, *homeostatic failure*, is depicted by Romero et al. (2009) as below the predictive homeostasis range and represents an animal’s inability to maintain or regulate normal mediator levels and death follows rapidly. A graphical representation of the reactive scope model in the absence of stress events is presented in Fig. 1 where the thresholds between the predictive homeostasis range, reactive homeostasis range, and homeostatic overload range are labeled *P*(*t*) and *R*(*t*) respectively. See Sect. 2.2 for further discussion of these values.

An important aspect of the reactive scope model is wear and tear: the sizes of the ranges associated with each classification can change temporarily or permanently in response to stress events. “Sizes” refers to the distance between the threshold values between the ranges on the mediator *y*-axis. The result of this wear and tear is that a raised mediator level can be tolerated by an animal for some period of time before homeostatic overload is induced, mimicking resilience, and that the same stress response may lead to homeostatic overload more rapidly in the future. According to Romero et al. (2009), “there are two ways to enter Homeostatic Overload: the concentration or level of the mediator extends beyond the normal reactive scope; or the concentration or level of the mediator remains in the Reactive Homeostasis range for an extended period.”

We introduce our model below by first discussing and giving a mathematical representation of stress events and schedules and then of the thresholds between three primary characteristic ranges of the reactive scope model.

### The stress schedule

Romero et al. (2009) defines a stress event as an unpredictable event in an animal’s environment and that a stress response is a physiological reaction to the event. They emphasize the exact behavior of the stress response is dependent on the mediator or behavior in question. Further, a stress event or response can have a variety of causes including allostatic overload as described by McEwen and Wingfield (2003), predation, or may even be the anticipation of a stressor event. Here, we disregard the distinction between a stimulus event and a response and use the term *stress event* to refer to any change in a physiological mediator level regardless of whether the cause is an environmental stimulus, allostatic overload, or psychological cause. To describe a mediator level during a stress event or series of stress events, we use the *stress schedule* as described by

1 $$\begin{aligned} s(t) = \sum _{i=1}^n s_i\psi _{\mu _i,\sigma _i}(t) \end{aligned}$$

where *n* is the number of stress events being considered, $s_i$ is the magnitude of the stress response for the $i^{\textrm{th}}$ stress event, $\psi _{\mu _i,\sigma _i}$ is a function that captures the desired behavior of the stress event (e.g. up- and down-regulation time, increase and decrease rates, etc.), and $\mu$ and $\sigma$ are parameters that dictate the time and duration of the event. It should be noted that different mediators will respond differently in any given situation and that a stress event and subsequent stress schedule are meant to capture the behavior of a single mediator over time. Thus, if several mediators are being considered, their respective stress schedules should be plotted on separate axes.

**Fig. 2:**
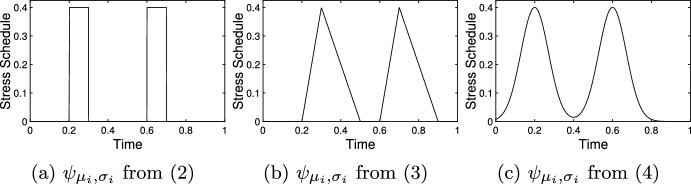
(**a**) The form of the stress schedule is given in (2). Stress schedules where $s_1=s_2=0.4$, $\sigma _1=\sigma _2=0.1$, $\mu _1=0.2$, and $\mu _2=0.6$ using different forms for $\psi$. These schedules are presented independently from consideration of physiological mediator, so the values on the independent axis are meaningless here. **b** With $\alpha =3$. Note that each event starts at the appropriate $\mu$ value. **c** The value of $\mu$ denotes the mid-time of the event

We provide three possible forms for $\psi _{\mu _i,\sigma _i}$ below and their graphs are shown in Fig. 2. First, the graphical model provided by Romero et al. (2009) describes stress events that are best presented using

2 $$\begin{aligned} \psi _{\mu _i,\sigma _i}(x) = {\left\{ \begin{array}{ll} 1 &{} \mu _i< x < \mu _i+\sigma _i \\ 0 &{} \text {otherwise} \end{array}\right. }. \end{aligned}$$

where $\mu _i$ and $\sigma _i$ are strictly positive constants. Here, $\mu _i$ would represent the beginning of the $i^{\textrm{th}}$ stress event and $\sigma _i$ indicates the duration of the event. This form of $\psi _{\mu _i,\sigma _i}$ is useful for demonstrative purposes but is likely to provide an oversimplified representation of the stress response as it will result in mediator levels up- and down-regulating instantaneously.

Rich and Romero (2005) recorded corticosterone levels in birds undergoing induced stress. The data presented suggests that a stress response commences at the initiation of a stress event and is up-regulated throughout the stress event. At the conclusion of the stress event, the mediator is down-regulated over a period of time that is longer than the stress event. Such a process is better represented using $\psi _{\mu _i,\sigma _i}$ given by

3 $$\begin{aligned} \psi _{\mu _i,\sigma _i}= {\left\{ \begin{array}{ll} \frac{1}{\sigma _i}(x-\mu _i) &{} \mu _i< x< \mu _i+\sigma _i\\ \frac{1}{\sigma _i-\alpha \sigma _i}(t-\mu _i-\sigma _i) &{} \mu _i+\sigma _i< x < \mu _i+\alpha _i \sigma _i \end{array}\right. } \end{aligned}$$

where $\alpha >0$ and $\alpha \ne 1$. The value of $\mu _i$ should coincide with the beginning of the $i^{\textrm{th}}$ stress event (or can be adjusted if the delay period between the stimulus and response is known) and $\sigma _i$ represents the duration of the stress event or up-regulation period. The parameter $\alpha$ represents how much longer down-regulation of the mediator takes. For example, Rich and Romero (2005) showed that the down-regulation of CORT is 3 to 4 times as long as the up-regulation.

Other options may be more appropriate for the $\psi$ depending on circumstances. For example,

4 $$\begin{aligned} \psi (x) = e^{-\left( \frac{t-\mu _i}{\sigma _i}\right) ^2} \end{aligned}$$

where $\mu _i$ represents the mid-time of the stress event would provide a continuously differentiable version of the stress event that may more accurately capture the nature of the up- and down-regulation. However, $\sigma _i$ in Eq. (4) does not directly represent the duration of the stress event nor response. That is, larger values of $\sigma _i$ would imply a longer duration but neither the event nor the response would have a duration equal to $\sigma _i$. Figure 2 compares the three descriptions for stress events provided here.

### Wear and tear of the reactive scope

A defining feature of the reactive scope model is what (Romero et al. 2009) referred to as “wear and tear.” Unlike the use of “wear and tear” to refer to the long-term, slow accumulation of damage and aging (McEwen 1998), wear and tear is the idea that repeated or prolonged stress responses may cause a decrease in the size of an animal’s reactive homeostasis range during the stress response, thereby making it possible for future stress events of the same magnitude to lead to homeostatic overload. We model this behavior by changing the threshold curves between the characteristic ranges of the reactive scope model.

As the reactive scope model undergoes change in response to physiological mediator levels, we begin by describing the mediator level under consideration at time *t* by *y*(*t*) where

5 $$\begin{aligned} y(t) = a_s \sin (b_s t) + a_c \sin (b_c t) + s(t) + y_c, \end{aligned}$$

$a_s$ and $a_c$ are amplitude the of seasonal and circadian variation respectively, $b_s$ and $b_c$ are period of seasonal and circadian variation respectively, $y_{c}$ is the basal level of the physiological mediator (and must be chosen large enough to ensure that *y*(*t*) is nonnegative), and *s*(*t*) is the stress schedule as defined in (1). Thus, the mediator level *y*(*t*) is driven both physiological factors and the stress schedule.

Next, we describe the initial threshold curve, *P*(*t*), between the predictive homeostasis range and reactive homeostasis range by

6 $$\begin{aligned} P(t) = a_s \sin (b_s t)+a_c + y_c + y_{\textrm{act}}\end{aligned}$$

where $a_s$, $b_s$, $a_c$, $y_c$ are as above and $y_{\textrm{act}}$ represents changes in mediator level due to predictable activity.

We then denote the boundary between reactive homeostasis and homeostatic overload by *R*(*t*) and describe the hypothesized wear and tear of the reactive scope range by allowing *R*(*t*) to change in response to *y*(*t*). Since the reactive scope model assumes that the reactive scope range will return to its pre-stimulus size if the stress event fails to induce homeostatic overload, we introduce a new quantity, *M*(*t*), to represent the maximum value that *R*(*t*) can recover to at the conclusion of a stress event. According to Romero et al. (2009) with our notation, $R(t) \le M(t)$ for all *t*, *R*(*t*) should decrease temporarily if $P(t)< y(t) < R(t)$, should decrease permanently if $y(t)>R(t)$, and increases to *M*(*t*) when $y(t) < P(t)$. Further, *M*(*t*) decreases permanently when $y(t)>R(t)$. We let $\theta (x)$ denote the Heaviside function

7 $$\begin{aligned} \theta (x) = {\left\{ \begin{array}{ll} 0 &{} x \le 0 \\ 1 &{} x>0 \end{array}\right. }. \end{aligned}$$

Then,

8 $$\begin{aligned} \frac{d M}{dt}&= -r_1 \theta (y(t)-R(t)) \nonumber \\ \frac{dR}{dt}&= -r_2 \theta (y(t)-P(t))+r_3\theta (P(t) -y(t))\theta (M(t)-R(t)) \end{aligned}$$

where $M(0)=R(0)=M_0$; $r_1$, $r_2$, and $r_3$ are all strictly positive; and $r_1 \le r_2$. The system given in (8) mimics figures given by Romero et al. (2009) by letting $r_1=r_2=r_3$ as is shown in Fig. 1. In the absence of a stress event, $M(t) = R(t)$ for all *t*-values and their curves overlap. Here, $a_s=0.24$, $a_c=0.02$, $y_c = 0.3$, $y_{\textrm{act}}=0.01$. Note that the homeostatic failure range would appear below the predictive homeostasis range but is omitted in subsequent figures for simplicity. For the sake of this work, we assume $r_1=r_2=r_3$ unless otherwise specified. In Sect. 5.1 we provide several possible additions to the base model presented here and focus on the characteristics of the basic model for now.

**Fig. 3:**
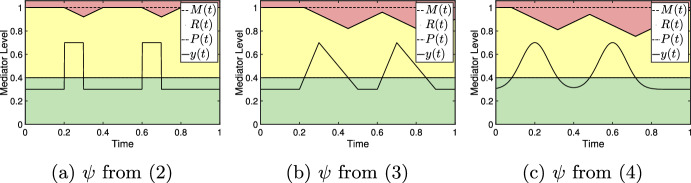
Mediator levels and homeostatic ranges within the reactive scope model. Since no particular mediators are being presented, the value 1 on the dependent axis would correspond to the mediator level of maximal effect. Similarly, values on the independent axis can be viewed as a proportion of the duration of an experiment. **a**–**c** A stress schedule where $s_1=s_2=0.4$, $\sigma _1=\sigma _2=0.1$, $\mu _1=0.2$, and $\mu _2=0.6$. The ranges are colored in correspondence to those in Fig. 1. **b** with $\alpha =3$. Note that each event starts at the appropriate $\mu$ value. **c** The value of $\mu$ denotes the mid-time of the event

To demonstrate the characteristics of the model, we consider a few examples independent from any particular experiment or mediator level. We set independent axes to scale from 0 to 1 where 1 can be thought of as the level of maximal effect (similar to $C_{100}$ in pharmacology (Derendorf et al. 2020)). Similarly, dependent axes are scaled to 1 to indicate experiment duration and we continue this convention unless otherwise noted. Figure 3 shows several representations of the reactive scope model along with various choices of $\psi _{\mu _i,\sigma _i}$. For each choice of $\psi _{\mu _i,\sigma _i}$, the length of time for which $y(t)>P(t)$ is different resulting in different behaviors in *R*(*t*). Since $y(t)<R(t)$ for all *t* in each graph, $M(t) = M_0$ for each graph.

For Fig. 3, and all remaining depictions of the model, the homeostatic failure range is removed to focus on the relevant portions of the figures. Further, with the exception of the figures in Sect. 3, figures throughout this work are meant to depict the qualitative relationships between an arbitrary physiological mediator and the homeostatic overload (red) and reactive homeostasis (yellow) ranges. As such, the scaling of the axis is effectively arbitrary and irrelevant.

**Fig. 4:**
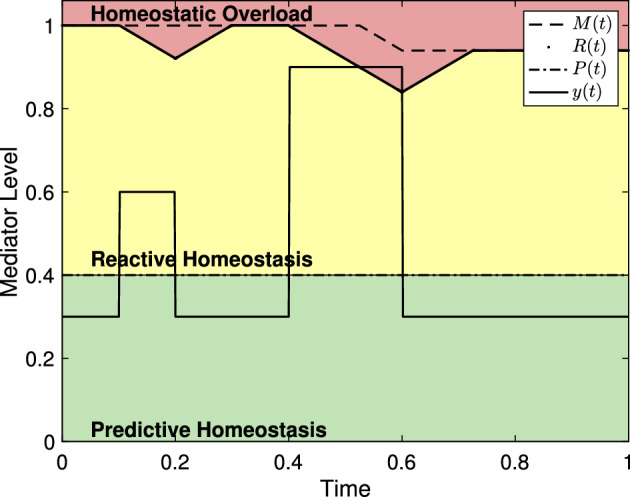
The reactive scope model with mediator levels driven by a stress schedule with two stress events. Here, $s_1 = 0.3$, $\mu _1 =0.1$, $\sigma _1=0.1$, $s_2 = 0.6$, $\mu _2 = 0.4$, and $\sigma _2 = 0.2$. The first stress event does not cause homeostatic overload, so the value of *M*(*t*) stays fixed while *R*(*t*) decreases then recovers to the value of *M*(*t*). During the second stress event, the raised value of *y*(*t*) causes *R*(*t*) to decrease below *y*(*t*) leading to homeostatic overload resulting in a permanent decrease in *M*(*t*)

Figure 4 shows reactive scope along with mediators driven by a stress schedule with two stress events. Since the first stress event does not cause homeostatic overload, the value of *R*(*t*) is temporarily decreased but *M*(*t*) does not. Thus, *R*(*t*) recovers to the original maximum value $M_0$. The second stress event lasts long enough for the reactive scope to decrease leading to homeostatic overload without the mediator level increasing further. As a result, the value of *M*(*t*) decreases permanently. Thus, the threshold value *R*(*t*) can never recover to the original value $M_0$.

**Fig. 5:**
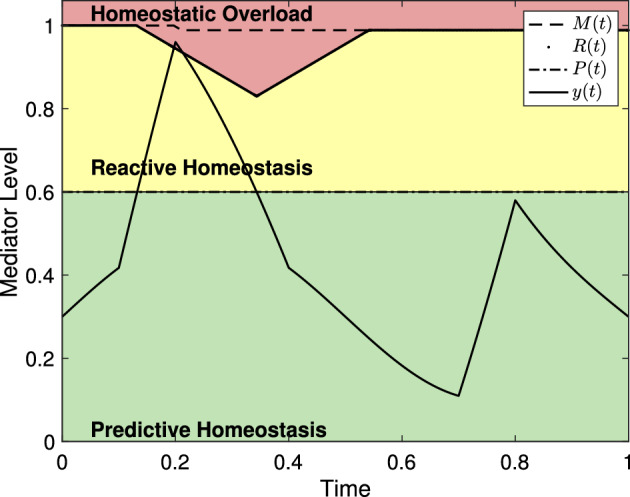
Reactive scope with mediator driven by a stress schedule and circadian variation. Here $a_c = 0.2$, $b_c = 2\pi$, $y_c = 0.3$, and $y_{\textrm{act}}= 0.1$. For the stress schedule, $s_1 = s_2 = 0.47$, $\sigma _1 = \sigma _2 = 0.1$, $\mu _1 = 0.1$, and $\mu _2 = 0.7$. $\psi _{\mu _i,\sigma _i}$ is taken from (3) with $\alpha = 3$

Figure 5 shows reactive scope along with mediators driven by a stress schedule and undergoing circadian variation. The magnitude of the stress event is the same in both, but the first causes homeostatic overload because it occurs when basal levels are at their peak. As a result *M*(*t*) is permanently decreased. Despite having the same magnitude, the second stress event does not cause the mediator level to grow above the predictive homeostasis range so *R*(*t*) does not decrease.

## Replication of experimental results

A number of empirical studies have interpreted results using the reactive scope model (Aguilar et al. 2016; Beattie et al. 2023a; Charpentier et al. 2018; Crespi et al. 2013; Eguizábal et al. 2022; Gabriel et al. 2018; Houtz et al. 2022; Howell and Sanchez 2011; Leishman et al. 2022; Lima et al. 2022; Pahuja and Narayan 2021; Roast et al. 2019; Romero 2012; Schoenle et al. 2018). However, only a few studies, including Beattie et al. (2023b), Gormally et al. (2019b), Gormally et al. (2019a), DuRant et al. (2016b), and DuRant et al. (2016a), have been conducted with the explicit purpose of investigating the reactive scope model. As these studies include the use of accepted situations known to elicit a stress response, regular measurements of physiological mediators associated with the stress response, and provide ample information for the recreation of results using the model, we focus on the latter group here. The studies predominantly focus on corticosterone levels in birds. Corticosterone (CORT) is the primary avian glucocorticoid (Holmes and Phillips 1976), though catecholamines have also been found to be a valid indicator of the stress response and a good fit for the reactive scope model (Fischer and Romero 2013). We replicate some of the results related to CORT shown by DuRant et al. (2016a) in which CORT levels were measured four times over a 25 day period. Since the measurements in the study where too infrequent to capture circadian variation, we will base basal levels on those found by Breuner et al. (1999) where basal CORT levels in Gambel’s White-Crowned Sparrow (*Zonotrichia leucophrys gambelii*) were found to vary daily with a maximum basal value less than 30 ng/ml occurring just before the active period. We will assume a similar pattern holds for the house sparrows (*Passer domesticus*) used by DuRant et al. (2016a). We will ignore seasonal variation due to the relatively short time period for the experiment.

To mimic the above mentioned circumstances, we set $y_c = 12$ ng/ml, $a_s = 0$ (and therefore $b_s = 0$), $a_c = 6$ ng/ml, and $b_c = 2\pi$. Figures provided by DuRant et al. (2016a) indicated that CORT levels increased from day 1 to day 12 of the experiment, then declined until day 25 which was the day of the last blood sample. Accordingly, we will use a single stress event with $\psi$ in the form of (3) with $s_1 = 140$ ng/ml, $\mu = 1$ day, $\sigma = 11$ days. CORT levels had not returned to normal at the time of the last blood sample, so we set $\alpha = 4$ assuming the pattern presented continues.

**Fig. 6:**
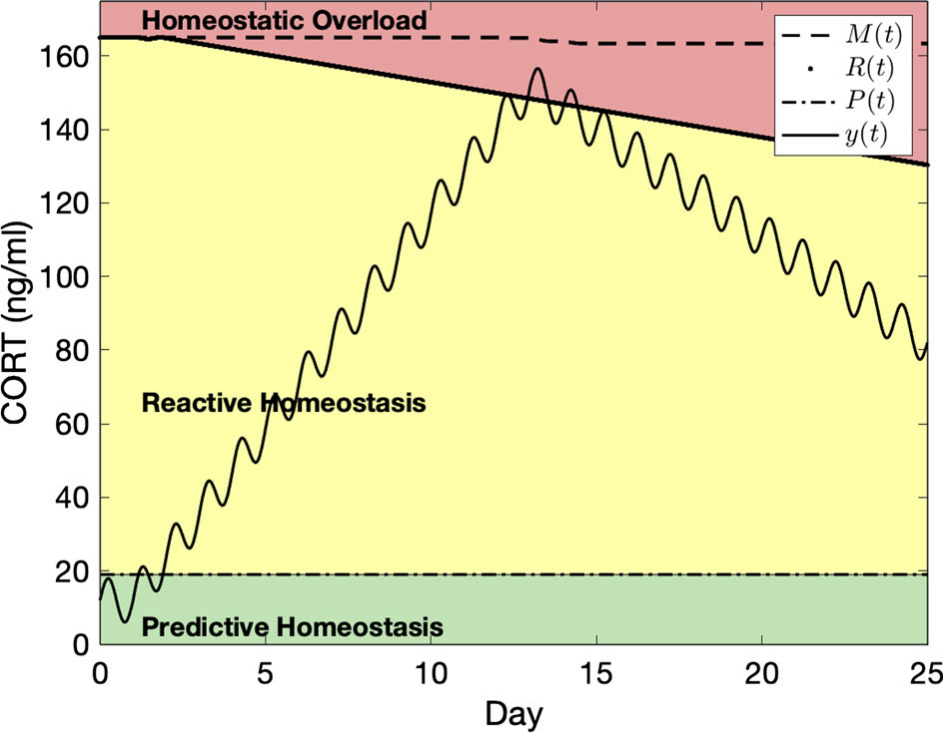
A recreation of the mediator levels for birds with artificially elevated CORT levels from DuRant et al. (2016a). The oscillation is caused by the circadian variation in *y*(*t*)

DuRant et al. (2016a) indicated that the birds with artificially elevated CORT levels had decreased wound healing ability indicating that this group may have experienced homeostatic overload as a result of the experiment (though the authors note the results were not statistically significant). As it is not possible to estimate $y_{\textrm{act}}$, $M_0$, $r_1$, $r_2$, and $r_3$ from the information given, we determine these values to be $y_{\textrm{act}}= 1$, $M_0 = 165$ ng/ml and $r_1=r_2=r_3=1.5$ by inspection. The model output with these variables is shown in Fig. 6.

Our goal here is not to validate nor reject the work of DuRant et al. (2016a). Neither do we claim that the ability to use the mathematical framework for reactive scope presented here to reproduce empirical work validates the mathematical formulation of the model nor the conceptual framework. Rather, we present this recreation in hopes of showing the flexibility and potential of the mathematical framework.

### Analysis of the model

The reactive scope model is entirely driven by the mediator level *y*(*t*) which is, in turn, driven by seasonal and circadian variation and the stress schedule. The system given in (8) predicts no change in *R*(*t*) nor *M*(*t*) unless $y(t) > P(t)$. That is, in the absence of stress there is no change in reactive scope. (In Sect. 5.1 we consider a version of the model that allows *R*(*t*) and *M*(*t*) to decrease over time.) In the presence of a stress event and elevated mediator levels, the characteristics of the mediator largely determine the behavior of the system. A simple, stationary pendulum serves as a good analogy for this system. Left alone, the pendulum will not undergo any change. If the pendulum is perturbed in some way, it will react but the characteristics of that reaction will depend on the specifics of the force applied. Much like the pendulum, mediator levels can be manipulated in a variety of ways.

**Fig. 7:**
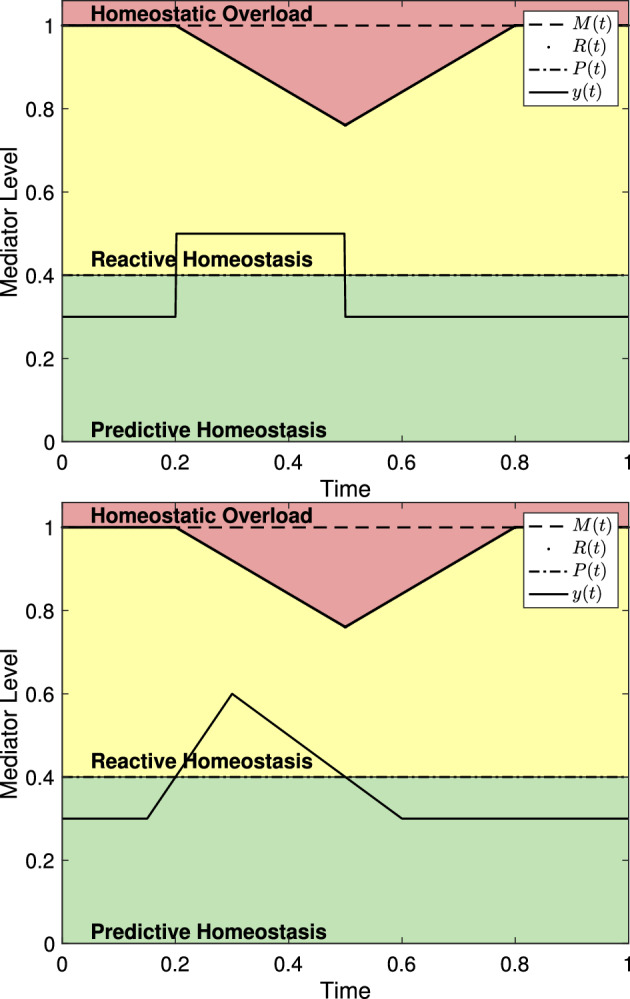
Two versions of the reactive scope system as presented in (8) using different $\psi _{\mu _i,\sigma _i}$ functions. In both, $y(t)>P(t)$ for $0.2< t< 0.5$ and *R*(*t*) is the same for both. (*left*) $\psi _{\mu _i,\sigma _i}$ as given in (2) with $\mu = 0.2$, $\sigma = 0.3$, and $s_1 = 0.2$. (*right*) $\psi _{\mu _i,\sigma _i}$ as given in (3) with $\mu = 0.15$, $\sigma = 0.15$, and $\alpha = 3$

For a minor stress event, one for which the mediator level *y*(*t*) only temporarily exceeds *P*(*t*) and does not exceed *R*(*t*), the choice of $\psi _{\mu _i,\sigma _i}$ has no significant impact on the predictions of the model. The system in (8) predicts that *R*(*t*) will decrease while *y*(*t*) exceeds *P*(*t*), but that decline is in no way proportional to the magnitude of *y*(*t*) nor the rate of increase of *y*(*t*). Figure 7 shows the reactive scope system with stress events using different $\psi _{\mu _i,\sigma _i}$ and different magnitudes, but for each the the length of the time interval in which $y(t)>P(t)$ is the same. All figures in this section are given with unit-less axes that can be assumed to represent the proportion of the mediator experiment time. Thus, the model is not sensitive to the choice of $\psi _{\mu _i,\sigma _i}$ if homeostatic failure is never induced.

**Fig. 8:**
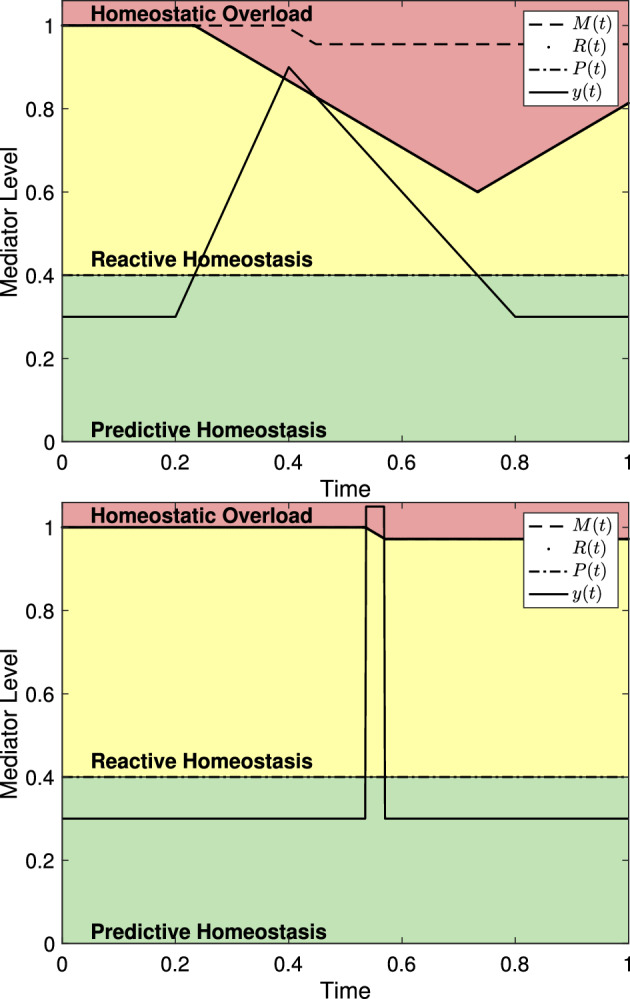
Two versions of the reactive scope model with homeostatic overload. In both, $y(t)>R(t)$ for approximately 0.033834 time units and, therefore, *M*(*t*) decreases by $0.033834r_1$ (with $r_1 = 0.8$ here). (*left*) $\psi _{\mu _i,\sigma _i}$ as in (3) with $s_1 = 0.6$, $\mu = 0.2$, $\sigma = 0.2$, and $\alpha = 3$. (*right*) $\psi _{\mu _i,\sigma _i}$ as in (2) with $\mu \approx 0.39474$, $\sigma \approx 0.42857$, and $s_1 = 0.75$

Similarly, if a stress event is significant enough for *y*(*t*) to exceed *R*(*t*), then only the length of the time interval during which $y(t)>R(t)$ causes *M*(*t*) to decline. Two stress events with different characteristics and magnitudes will cause the same decline in *M*(*t*) if the time spent with $y(t)> R(t)$ is the same. However, stress events with different characteristics will cause the mediator level to spend different amounts of time in the reactive homeostasis range leading to different declines in *R*(*t*). Figure 8 shows two versions of the reactive scope model with both spending the same amount of time in homeostatic overload.

**Fig. 9:**
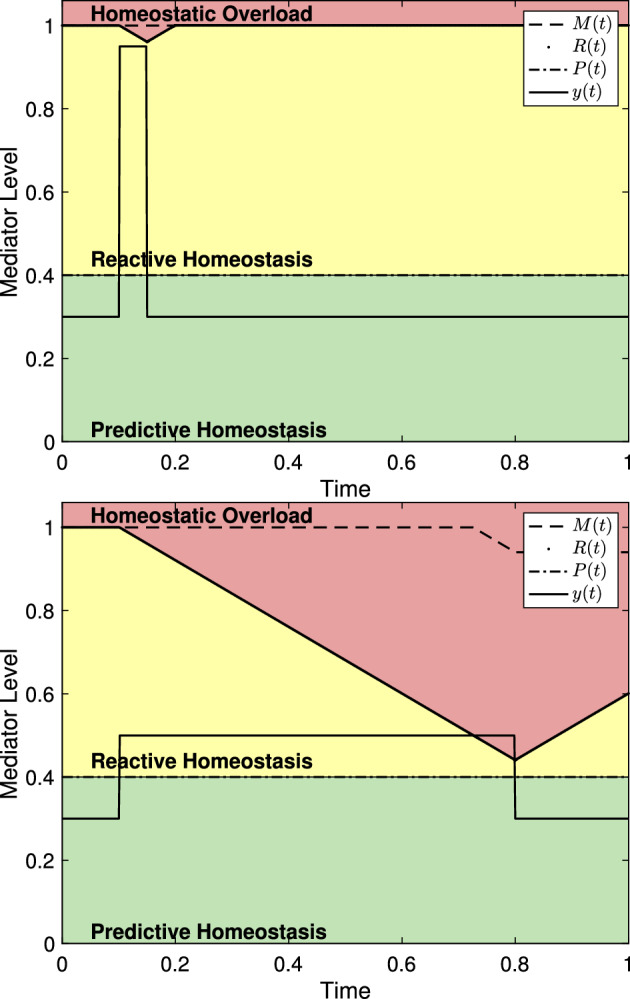
The reactive scope model shown with mediator levels of varying magnitude and duration. (*left*) A mediator level with $s_1 = 0.65$, $\mu =0.1$, and $\sigma =0.05$ does not cause homeostatic overload. (*right*) A mediator level with $s_1=0.2$, $\mu = 0.1$ and $\sigma = 0.7$ causes homeostatic overload

As Fig. 8 suggests, the reactive scope model places significant emphasis on the duration of a stress event. Figure 9 shows two versions of the system in (8) with stress events of different magnitude and duration. As can be seen, a severely elevated mediator level may not lead to homeostatic overload if its duration is short enough, while having mediator levels elevated to the reactive homeostasis range for a prolonged period may lead to homeostatic overload.

**Fig. 10:**
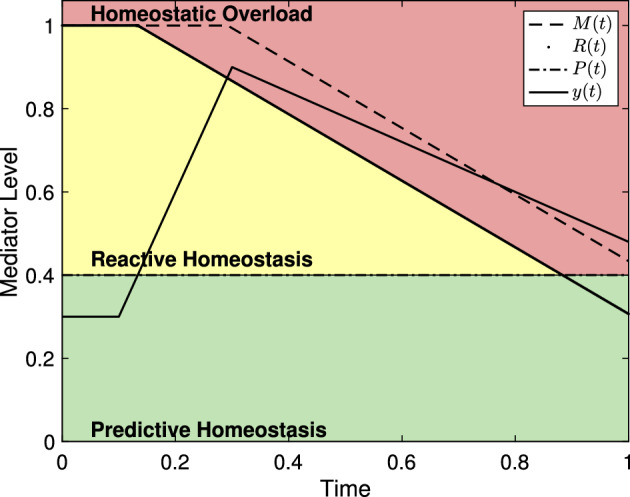
A physiological mediator whose down-regulation occurs at a lesser rate than $r_2$ cannot escape homeostatic overload

The rate of down-regulation of a physiological mediator may provide some insight into $r_2$, the rate of decrease of *R*(*t*). If an animal has a down-regulation rate that is greater than $r_2$, then that animal would not be able to escape homeostatic overload once begun. Figure 10 describes such an event. This would imply that $r_2$ must be greater than the down-regulation rate for a given mediator.

## Verification of the model

As the reactive scope model predicts a decline in the reactive homeostasis range during a stress response, and a decline in the maximal reactive homeostasis during a significant stress response, the model may be verified if repeated stress events induce homeostatic overload over shortened time intervals.

**Fig. 11:**
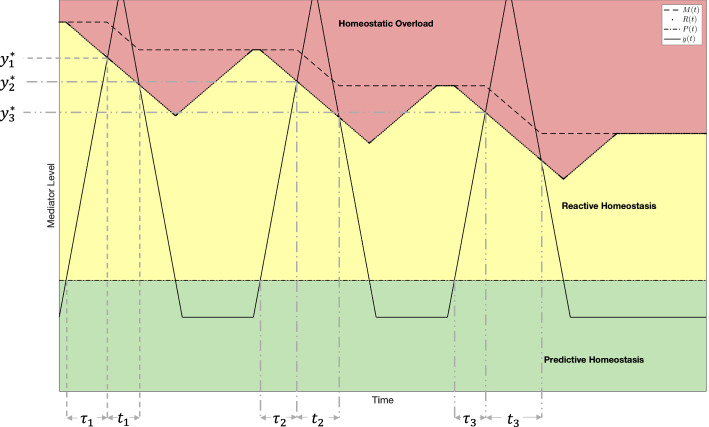
A graphical depiction of the stress events as described in Sect. 4 with time ranges and corresponding mediator levels labeled. For the purposes of this graph $r_1 = r_2 = r_3 = 2$

We consider a series of stress events that each cause a rise in *y*(*t*) significant enough to induce homeostatic overload and spaced far enough apart to allow the value of *R*(*t*) to recover to the value of *M*(*t*) and assume that $r_1 = r_2 = r_3 = r$. See Fig. 11. For the first stress event, we let $\tau _1$ denote the length of time during which $P(t)<y(t)<R(t)$. During this time span, *R*(*t*) will decline as *y*(*t*) continues to increase until their values intersect. Denote the mediator level when the curves intersect by $y_1^*$. Since we know that *R*(*t*) decreased by $r\tau _1$ during this time interval, we know that $M_0 = y_1^*+r\tau _1$. Since the first stress event induced homeostatic overload, the value of *M*(*t*) will decrease. If the period of homeostatic overload is denoted by $t_1$, then $M(t) = M_0 - rt_1$ at the conclusion of the period of homeostatic overload.

Similarly, during the second stress event *R*(*t*) declines by $r\tau _2$ until it intersects *y*(*t*) at the mediator level $y_2^*$ and homeostatic overload commences. We denote the duration of homeostatic overload by $\tau _2$ and note that *M*(*t*) declines by $r \tau _2$ during this time. Thus, $y_2^*+r\tau _2 = M_0-rt_1$. That is

9 $$\begin{aligned} M_0 = y_2^*+rt_1+r\tau _2. \end{aligned}$$

We know from the first stress event that $M_0 = y_1^*+r\tau _1$, thus

10 $$\begin{aligned} y_2^*+r\tau _2&= y_1^*+r\tau _1 - rt_1\end{aligned}$$

11 $$\begin{aligned} rt_1 -r\tau _1 + r\tau _2&= y_1^*-y_2^*\end{aligned}$$

12 $$\begin{aligned} r&= \frac{y_1^*-y_2^*}{t_1 - \tau _1 + \tau _2}. \end{aligned}$$

Repeating this pattern for another stress event, we may find that

13 $$\begin{aligned} M_0 = y_3^* + rt_1+rt_2 + r\tau _3. \end{aligned}$$

Combining (9) with (13), we find that

14 $$\begin{aligned} y_3^* +rt_1+rt_2 + r \tau _3&= y_2^* + r t_1 + r\tau _2\end{aligned}$$

15 $$\begin{aligned} rt_2+r\tau _3 - r\tau _2&= y_2^*-y_3^*\end{aligned}$$

16 $$\begin{aligned} r&= \frac{y_2^*-y_3^*}{t_2 - \tau _2 + \tau _3}. \end{aligned}$$

We can continue this pattern to find that

17 $$\begin{aligned} r = \frac{y_i^*-y_{i+1}^*}{t_i+\tau _{i+1}-\tau _i} \end{aligned}$$

meaning that *n* stress events can be used to compute *r* a total of $n-1$ times. Hence, if the value of the ratio given in (17) is consistent across multiple stress events, then the values of the maximal reactive homeostasis and reactive homeostasis must be declining linearly as predicted.

There are several benefits to this approach. First, the actual value of $M_0$ need not be known. Indeed, since the reactive scope model predicts a decline in *M*(*t*) in the event of significant stress, any wild-caught animals are likely to have varying levels for $M_0$. Additionally, any individual animal being examined need only endure three stress responses for a verification and any further stress responses can be used for further verification. Finally, even if the values found are inconsistent, their behavior should indicate how to adjust the decline of reactive scope.

## Discussion

The mathematical formulation provided in (8) is meant to mimic the description of reactive scope as described by Romero et al. (2009) as much as possible. In this section, we discuss some potential additions to the model as well as weaknesses of the model.

### Potential additions to the model

**Fig. 12:**
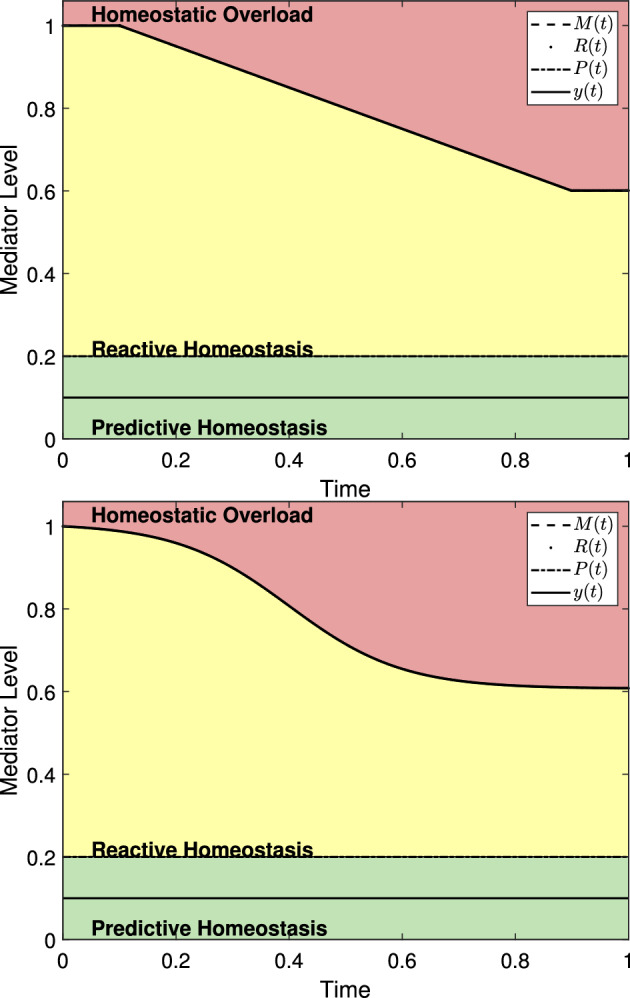
The reactive scope model shown over an animal’s lifetime normalized to $t=1$ with senescence included and no stress events. (*left*) The function *A*(*t*) as described in (20) is used with $a_1=0.1$, $a_2 =0.9$, $p_{a_1} =1$, and $p_{a_2} =0.6$. (*right*) The function *A*(*t*) as described in (22) is used with $p_i = 1$, $p_f = 0.6$, $\bar{A} = 0.4$, and $k=10$. Due to the absence of stress, $M(t) = R(t)$ for all *t*-values and the curves overlap

The reactive scope model as presented in (8) largely describes stress responses in short-term situations. However, an animal’s response to stress is likely to change throughout its lifetime (Andrews et al. 2017; Barbi et al. 2018; Haussmann and Heidinger 2015). Senescence may be included in (8) via

18 $$\begin{aligned} \frac{d M}{dt}&= -r_1 \theta (y(t)-R(t)) - A(t) \nonumber \\ \frac{dR}{dt}&= -r_2 \theta (y(t)-P(t))+r_3\theta (P(t)-y(t)) \theta (M(t)-R(t))-\theta (R(t)-M(t))A(t) \end{aligned}$$

where the function *A*(*t*) describes the decline in the maximum threshold for homeostatic overload, *M*(*t*), over time. Figure 12 shows two possible examples of (18) using different forms for *A*(*t*). As an example, we may define

19 $$\begin{aligned} A(t) = {\left\{ \begin{array}{ll} 0 &{} t\le a_1\\ \frac{p_{a_1}-p_{a_2}}{a_1-a_2} &{} a_1< t \le a_2\\ 0 &{} a_2 < t \end{array}\right. } \end{aligned}$$

to describe a linear decline between the ages $a_1$ and $a_2$ from an *M*(*t*) value of $p_{a_1}$ at age $a_1$ to a value of $p_{a_2}$ at age $a_2$. Here, if the time interval in question is an animal’s entire lifespan, than $p_{a_1} = M(0)$. An advantage of the form of *A* given by (19) is that it allows for simple determination of *M*(*t*) in the absence of stress events that cause homeostatic overload. That is, if $y(t)<R(t)$ for all *t* and we incorporate the desired initial conditions, then

20 $$\begin{aligned} M(t) = \int \frac{d M}{dt} dt = \int A(t) dt = {\left\{ \begin{array}{ll} M_0=p_{a_1} &{} t \le a_1 \\ \left( \frac{p_{a_1}-p_{a_2}}{a_1-a_2}\right) (t-a_1)+p_{a_1} &{} a_1< t \le a_2\\ p_{a_2} &{} a_2 < t \end{array}\right. }. \end{aligned}$$

This allows $M(a_1) = p_{a_1}=M_0$ and $M(a_2) = p_{a_2}$. This simplicity allows for the parameters of *A* to be set once known. However, the form of *A* given in (19) is not continuous and describes an abrupt change in the behavior of *M*(*t*) and *R*(*t*) that may be unrealistic in an animal.

To describe a more natural decline in *M*(*t*) and *R*(*t*), we may consider *M*(*t*) in the absence of stress events with as described by

21 $$\begin{aligned} M(t) = p_{i} - \frac{p_i-p_f}{1+e^{k(\bar{A}-t)}} \end{aligned}$$

where $p_i$ denotes the initial value for *M*(*t*), $p_f$ denotes the final value for *M*(*t*), *k* represents the slope of the transition from $p_i$ to $p_f$, and location parameter $\bar{A}$ represents the midpoint of the transition period from $p_i$ to $p_f$. For example, if an animal were to live for 100 days, begin its life with $M(0)=M_0$, end its life with $M(t) = 0.6M_0$, with *M*(*t*) declining roughly between days 10 and 90 then we would have $p_i = M_0$, $p_f = 0.6M_0$, and . A *k* value that gives the desired transition rate can be selected. Then we may take

22 $$\begin{aligned} A(t) = \frac{d}{dt} \left[ p_{i} -\frac{p_i-p_f}{1+e^{k(\bar{A}-t)}}\right] = -\frac{k(p_i-p_f)e^{k(\bar{A}-t)}}{\left( 1+e^{k(\bar{A}-t)}\right) ^2}. \end{aligned}$$

Figure 12 shows *M*(*t*) over an animal’s lifespan using the two choices for *A*(*t*) presented here in the absence of stress events. Since the inclusion of senescence in the reactive scope model requires additional parameters that would require observation over an animal’s full lifespan, we leave further discussion of the model with senescence and its verification to future work.

The version of the reactive scope model presented in Sect. 2.2 used the Heaviside function $\theta$ to “switch” the behavior of the system provided in (8) based on the mediator value, *y*(*t*), in relation to reactive scope threshold, *R*(*t*). It might be more appropriate for this switch to happen more gradually as *y*(*t*) exceeds *R*(*t*). A function that demonstrates this desired behavior is given by

23 $$\begin{aligned} \Theta _c(x) = \frac{1}{1+e^{-cx}} \text { for } c>0. \end{aligned}$$

As $c \rightarrow \infty$ in (23), the sigmoid function $\Theta$ approximates the Heaviside function given by (7). It should be noted that this update would still require the inclusion of the Heaviside function to ensure $R(t) \le M(t)$. That is, (8) should be updated to

24 $$\begin{aligned} \frac{d M}{dt}&= -r_1 \Theta _c(y(t)-R(t)) \nonumber \\ \frac{dR}{dt}&= -r_2 \Theta _c(y(t)-P(t))+r_3\Theta _c(P(t)-y(t)) \theta (M(t)-R(t)). \end{aligned}$$

**Fig. 13:**
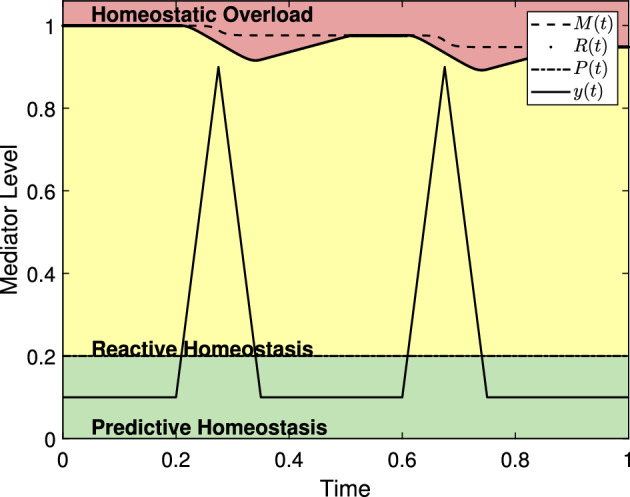
A demonstration of the reactive scope model using the sigmoid switching function as in (24). Notice that the value of *M*(*t*) decreases even though $y(t)<R(t)$ for all *t*. Here, $r_1=2.8$ while $r_2 =r_3 = 0.8$ and $c = -10$ to emphasize the change in *M*(*t*)

Figure 13 shows the reactive scope model as shown in (24). The only significant change is that the value of *M*(*t*) decreases while $y(t)<R(t)$ due to the low choice of $c = 10$ to emphasize this behavior. For a more realistic choice of *c* (e.g. $c>20$), the behavior of (24) is nearly indistinguishable from that of (8). Thus, the addition of this extra function does little to change the predictions of the model while adding a new parameter that must estimated in practice. Further, it is possible that the value of *c* may be different between the two equations in (24). Due to the additional complications introduced by the inclusion of $\Theta _c$ in the model, we will not consider it further here but have presented in an effort to consider alternatives.

### Limitations of the reactive scope model

There are limitations to the reactive scope model. There are various physiological mediators at play during any stress response. All such mediators work within their own homeostatic ranges and scales and may cause homeostatic overload at different levels (Romero et al. 2015). Thus, should a stress event lead to homeostatic overload, it may not be possible to determine which mediator at play left the reactive homeostasis range and triggered homeostatic overload. There is a growing consensus that glucocorticoids may be an imperfect measure of stress and that measuring more than one mediator is vital to understanding the stress response (Creel et al. 2013; Currie et al. 2010; Du et al. 2009; Jessop et al. 2013; Romero et al. 2015; Tomiyama et al. 2012). After a broad literature review, Dickens and Romero (2013) declared that there is no consensus on the endocrine profile of a chronically stressed wild animal. It was hypothesize by Gormally et al. (2019b) that these variations may be at least partially explained by differences in experimental design pertaining to species and life-history stage.

Further, the values of $r_1$, $r_2$, and $r_3$ and even $M_0$ may be different between various mediators, species, and individuals. As a result, it may be especially challenging to apply experimental results to any wild animal with an unknown life history. However, if general ranges can be established, the reactive scope model may provide valuable insight into wild populations undergoing shared chronic stress like pollution or habitat loss.

The boundary between the predictive homeostatic range and homeostatic range, described as *P*(*t*) in (6), represent a potential mathematical challenge since the boundary between reactive homeostasis and homeostatic overload, *R*(*t*), only declines when a mediator level exceeds *P*(*t*). Thus, determining values for $\tau$ as described in Sect. 4 requires being able to determine when this happens empirically. Further, simply capturing a wild-animal and transporting to a lab may be enough to alter the animal’s stress physiology (Dickens et al. 2009).

## Summary

The introduction of the reactive scope model by Romero et al. (2009) provided a novel framework for the study of stress responses in animals. A key feature of this framework is the inclusion of the reactive homeostasis range for mediator levels. Sustained mediator levels within the reactive homeostasis range may cause homeostatic overload via a degradation of the reactive homeostasis range, thereby allowing for a stress event late in an animal’s life history (or after other recent stress events) to cause homeostatic overload even when the level of response would not have been enough to cause homeostatic overload early in the animal’s life history (or without other recent stress events). To date, the reactive scope model has only been presented as a conceptual model.

Experimental efforts have been made to test the veracity of the reactive scope model (Beattie et al. 2023a; DuRant et al. 2016b, a; Gormally et al. 2019b, a). These experiments have attempted to observe the degradation of the reactive scope range by causing repeated stress events for different groups of wild birds and observing how recovery time between stress events impacts mediator levels in later stress events. Because the reactive scope model existed only as a conceptual model, experimenters could not use the model to determine the necessary duration of stress events to allow for the observation of the degradation of reactive scope, nor the necessary recovery times to allow reactive scope to recover.

We have provided a quantitative formulation of the conceptual reactive scope model. Several possible descriptors ($\psi _{\mu _i,\sigma _i}$) have been given that can be used to describe the desired curve shapes of mediator levels during stress responses. These descriptors can be combined with information previously collected for a particular mediator in a given species to describe the mediator level as a function of time with the inclusion of stress events (*y*(*t*) in (5)). Data already exists that can be used to estimate the parameters needed to accurately describe an animal’s mediator levels over times as they fluctuate daily or seasonally. Further, we have captured the dynamic nature of the reactive homeostasis range by providing a system of ordinary differential equations, (8), that describe how the thresholds between the mediator levels change in response to elevated mediator levels. More work must be done before the parameter values in (8) can be estimated. However, such experiments would either provide the necessary estimates or invalidate the current form of the reactive scope model. We have also provided several potential additions or adaptations to the reactive scope model including methods for the incorporation of senescence.
